# Next-generation immunotherapies for head and neck squamous cell carcinoma: targeting novel immune checkpoints

**DOI:** 10.1080/07853890.2025.2561223

**Published:** 2025-09-24

**Authors:** Jiaqi Tang, Huan Li, Yaodong He, Zihui Yang, Xinjie Yang, Jianhua Wei

**Affiliations:** State Key Laboratory of Oral & Maxillofacial Reconstruction and Regeneration, National Clinical Research Center for Oral Diseases, Shaanxi Key Laboratory of Stomatology, Department of Prosthodontics, School of Stomatology, The Fourth Military Medical University, Xi’an, Shaanxi, China

**Keywords:** HNSCC, immune checkpoint, cancer therapy, tumour microenvironment

## Abstract

**Objective:**

The purpose of this review is to explore eight novel immune checkpoints (PD-L2, B7-H3, VISTA, BTLA, TIM-3, LAG-3, TIGIT and GITR) in head and neck squamous cell carcinoma (HNSCC), including their biological properties, therapeutic potential and the possibility of combining them with other immune checkpoints (ICs) or emerging biotechnological approaches such as nanomaterials, oncolytic viruses (OVs) and tumour vaccines, with the aim of providing new treatment directions for HNSCC.

**Methods:**

We conducted a review and analysis of numerous studies in the past 5 years to understand the expression patterns, roles and the current state of related therapies for these eight novel ICs in HNSCC. We also examined the synergistic effects of combining these ICs with immune checkpoint inhibitors (ICIs), nanomaterials, OVs and tumour vaccines.

**Results:**

These novel ICs show unique expression and functions in HNSCC, offering new targets for overcoming tumour immune heterogeneity and resistance. Therapies targeting these ICs, such as monoclonal antibodies and small molecule inhibitors, have shown potential in preclinical studies. Moreover, combining these ICs with ICIs, nanomaterials, OVs and tumour vaccines has demonstrated enhanced antitumour effects, indicating broad application prospects.

**Conclusion:**

The eight novel ICs and their integration with emerging biotechnological approaches provide new hope for HNSCC treatment. Further research is needed to clarify the mechanisms of these novel ICs and optimize combination strategies to improve treatment precision and efficacy for HNSCC patients.

## Introduction

Head and neck cancers account for approximately 3.8% of all cancers globally, with over 95% of cases classified as head and neck squamous cell carcinoma (HNSCC) [1]. It is the sixth most common malignant tumour worldwide, with more than 500,000 new cases diagnosed annually [2]. The incidence of HNSCC is increasing at an alarming rate, with projections indicating a 30% rise by 2030 [3]. However, the 5-year survival rate of HNSCC patients has remained largely unchanged for three decades [3,4]. The standard treatment for advanced HNSCC is a combination of radiotherapy, chemotherapy and surgery. However, these approaches have limited efficacy and significantly impair quality of life [2,3,5]. This phenomenon is predominantly caused by the highly heterogeneous and immunosuppressive tumour microenvironment (TME), which also made HNSCC to be characterized as a ‘cold tumour’. Among all the mechanisms, immune checkpoint (IC) molecules play a crucial role in promoting the immune evasion of tumour cells. Tumours can evade the immune response by reducing the expression of co-stimulatory ligands or increasing the expression of co-inhibitory molecules of these molecules, thereby escaping immune responses [6] and thereby undermining the normal functionality of the immune system – a persistent and formidable challenge in the realm of immunotherapy [7].

The advent of immune checkpoint inhibitors (ICIs) has brought considerable optimism for the treatment of HNSCC. In contrast to traditional chemotherapy and radiotherapy, which indirectly target tumour cells, IC-targeted immunotherapy is regarded as a low-toxicity, high-affinity and highly specific treatment that harnesses the host immune system to prevent tumour escape [8]. In 2018, the pioneering discoveries of Dr James Allison and Dr Tasuku Honjo on cytotoxic T-lymphocyte antigen 4 (CTLA-4) and programmed death protein 1 (PD-1) led to a surge in research and clinical application of ICIs, resulting in a notable enhancement in anti-tumour efficacy in patients with advanced solid tumours. Nevertheless, the effectiveness of conventional ICIs (e.g. pembrolizumab, nivolumab) remains constrained, with a 5-year survival rate for HNSCC ranging from 40% to 60% [9].

### The limitations of current ICIs

**Figure 1.**
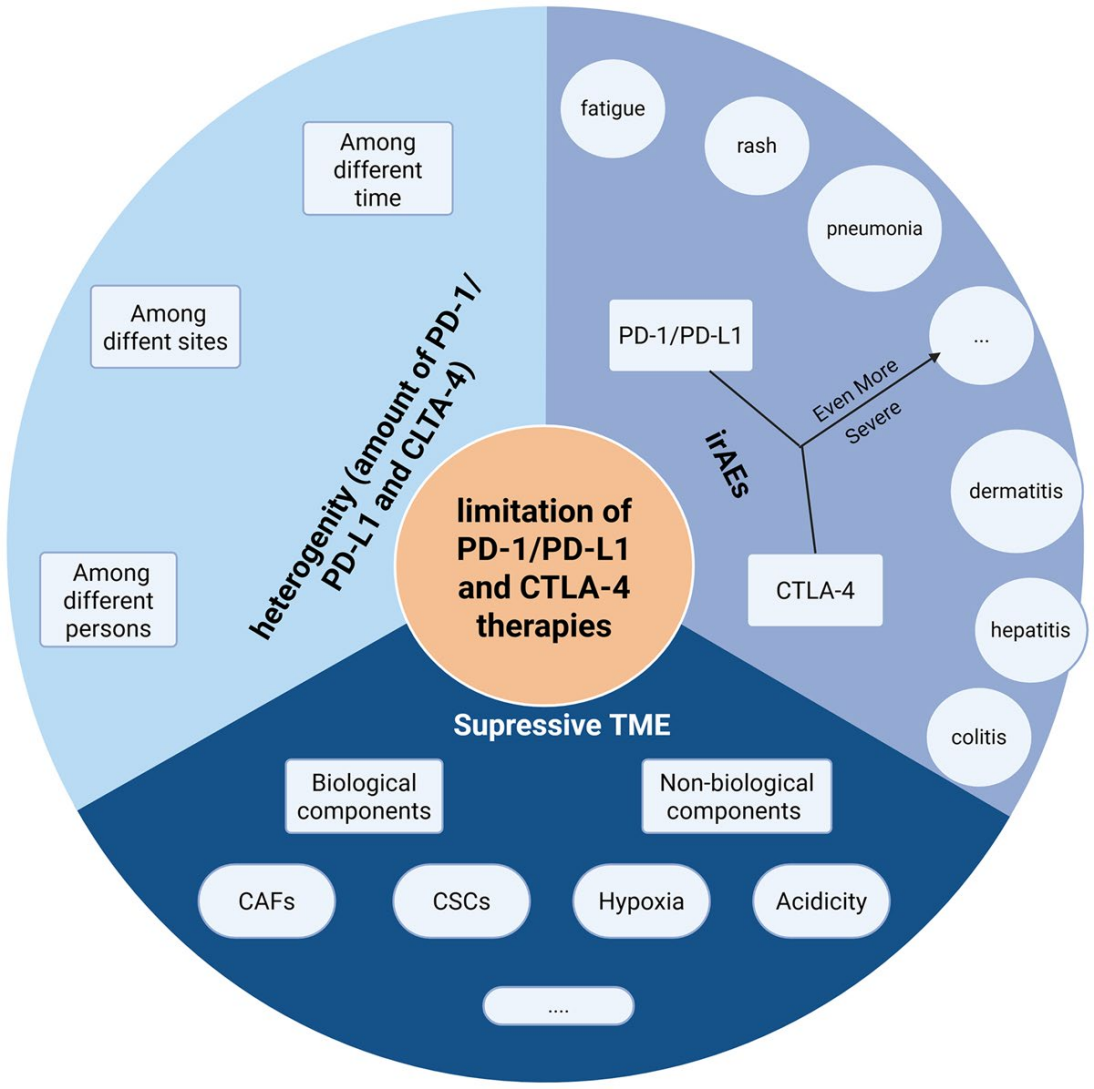
The figure schematically summarizes the major limitations of first-generation PD-1/PD-L1 and CTLA-4 blockade in HNSCC. Heterogeneity across time, anatomical sites and individuals undermines durable response; a suppressive tumour microenvironment (CAFs, CSCs, hypoxia, acidity) further blunts T-cell activity; respective and overlapping immune-related adverse events (fatigue, rash, pneumonia, colitis, etc.). Originally created by BioRender (https://www. BioRender. com).

However, there are still some limitations of PD-1/programmed cell death ligand 1 (PD-L1) and CTLA-4 related ICIs that hinders HNSCC therapies (Figure 1).

#### The heterogeneity of IC expression

Recent studies have revealed marked variability in PD-1 or CTLA-4 expression among individuals and across different tumour sites in patients with HNSCC, a disparity that may contribute to suboptimal responses to PD-1/PD-L1 and CTLA-4 therapies in a subset of these patients [10]. The dynamic fluctuations in PD-L1 expression levels may hinder the sustained efficacy of PD-L1 inhibitors in effectively blocking the PD-1/PD-L1 pathway [11].

#### The highly suppressive TME of HNSCC

The biological components, especially cancer-associated fibroblasts (CAFs) and cancer stem cells (CSCs), contribute to remodelling of the extracellular matrix and the sustained proliferation of cancer cells, respectively [3,4]. Non-biological factors, such as hypoxia and acidic environment also contribute to immune exhaustion. Consequently, only robust immunotherapy strategies can truly dismantle this distinctive tumour immunosuppressive microenvironment.

#### The increased irAEs

PD-1/PD-L1 therapies can elicit immune-related adverse effects, including fatigue, rashes and hypomagnesaemia, along with more severe reactions such as pneumonia and encephalitis, which may compromise treatment safety and tolerability [12]. Similarly, CTLA-4 inhibitors can cause serious irAEs like colitis, dermatitis and hepatitis [13]. Moreover, the combination of PD-1/PD-L1 therapies with CTLA-4 inhibitors may increase the risk of severe side effects, including significant skin and oral reactions, and not all patients benefit markedly from this combined approach [12].

These results highlight the need for the development of new treatment strategies and underscores the urgency of identifying new ICs for cancer immunotherapy. New IC molecules, including VISTA, TIM-3, LAG-3, TIGIT, programmed cell death ligand 2 (PD-L2), B7H3, BTLA and GITR, have been identified as potential targets for overcoming tumour heterogeneity and resistance, offering potential for more effective cancer immunotherapy [14]. Equally noteworthy are other checkpoints, such as GPR132, Siglec-15, ADAR1, CD3L1 and so on, which also demonstrate expansive research potential, but they have not entered clinical stage or had enough clinical researches [15]. The combination therapies involving these novel ICs – whether in conjunction with PD-1/PD-L1 and CTLA-4, or in collaboration with nanomaterials, oncolytic viruses (OVs) and tumour vaccines – have ignited a wave of research enthusiasm, offering new hope for immunotherapy in HNSCC. By simultaneously targeting multiple ICs, we can more comprehensively alleviate immune suppression, mitigate the resistance associated with single-target therapies, improve the durability of treatment and reduce the amount of irAEs, which ultimately enhances the safety and tolerability of treatment (Figure 1).

Therefore, in this review, we will provide an overview of the biological characteristics of eight ICs, their therapeutic implications in HNSCC and the prospective treatment possibilities when combined with three significant emerging biotechnological approaches.

## Introduction of novel ICs

**Figure 2.**
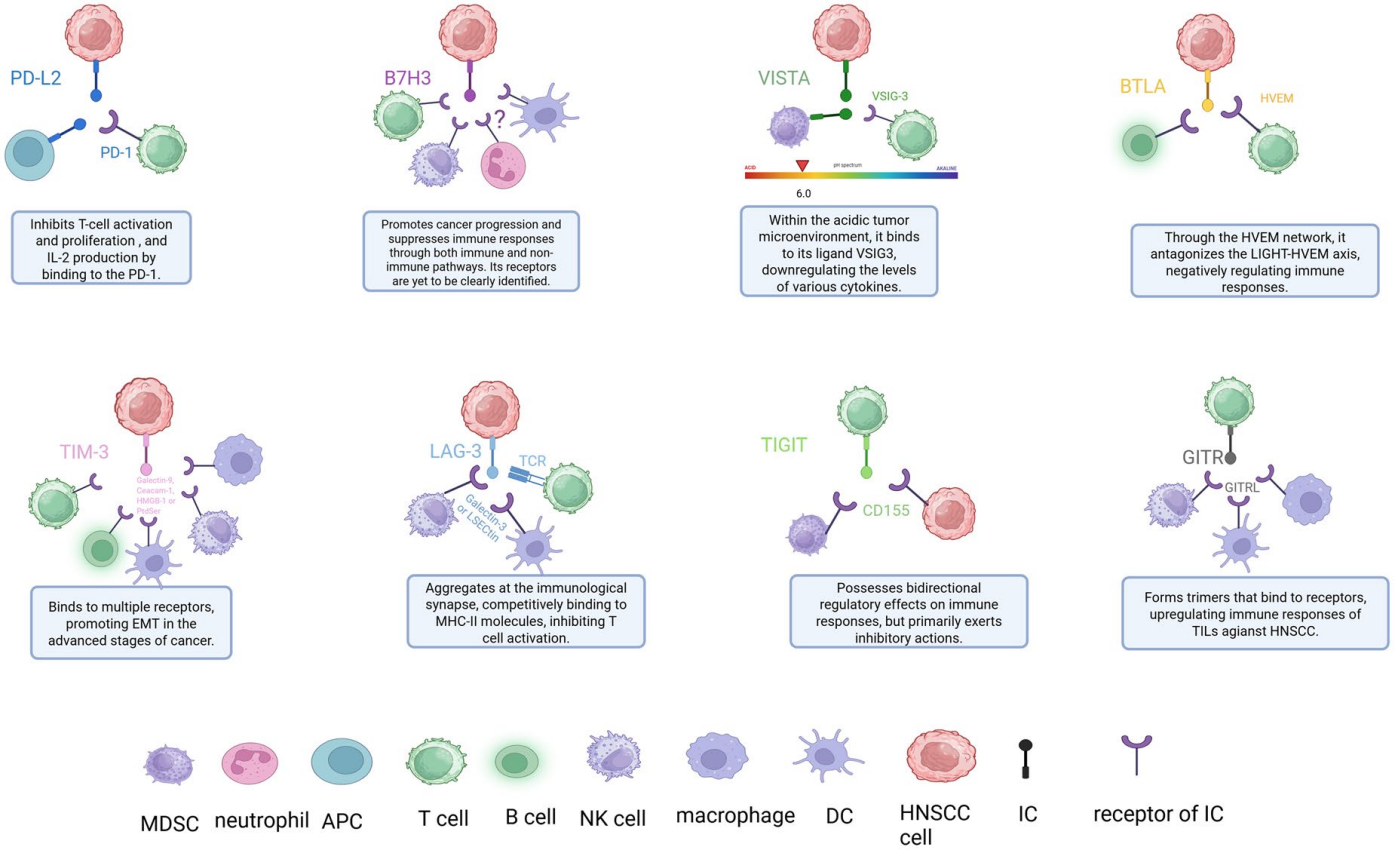
Landscape of emerging immune checkpoints and their ligand–receptor interactions in the tumour microenvironment of HNSCC. Each sub-panel illustrates the cellular source and spatial relationship of a single novel IC together with its cognate ligand(s), while simultaneously annotating the checkpoint’s functional consequence in the caption below. Originally created by BioRender (https://www. BioRender. com).

The identification of novel IC molecules following the discovery of PD-1/PD-L1 and CTLA-4 represents a significant advancement in the field of immunotherapy. These emerging ICs are playing an increasingly pivotal role in the treatment of HNSCC, providing additional therapeutic targets that may circumvent resistance to PD-1/PD-L1 and CTLA-4 inhibitors and enhance the therapeutic efficacy. These ICs are primarily categorized into two main families: the immunoglobulin superfamily (IgSF) and the Tumor Necrosis Factor Receptor (TNFR). The IgSF can be further delineated into CD28-B7 family, TIM family and other related families (Figure 2).

### Immunoglobulin superfamily

#### CD28-B7 family

##### PD-L2

PD-L2, also known as B7-DC, acts as a ligand for the IC molecule PD-1 and, in conjunction with PD-L1, plays a critical role in modulating T cell function and evading immune surveillance [16]. Compared to PD-L1, PD-L2 has a higher binding affinity for PD-1, with a binding constant two to six times greater than that of PD-L1. This increased affinity has been attributed to a tryptophan residue at position W110 in PD-L2 [17].

In tumour cells, PD-L2 is more widely expressed than PD-L1. For instance, in HNSCC, PD-L1 is predominantly expressed in antigen-presenting cells (APCs) and tumour cells, whereas PD-L2 is predominantly present in a broad range of immune cells, including macrophages and myeloid-derived mast cells. The proportion of PD-L2 expression is more than twice that of PD-L1 positivity [18]. PD-L2 suppresses T cell activation and proliferation by binding to the PD-1 receptor on the surface of T cells, while suppressing interleukin (IL)-2 production and promoting immune escape of tumours. Furthermore, there may be some interaction between PD-L2 and PD-L1 in tumours. For example, PD-L2 expression is increased in tumour-associated macrophages (TAMs), and the immune escape effect of PD-L2 in colorectal cancer is evident when PD-L1 function is inhibited [16]. This interaction can be further investigated in HNSCC.

The role of the hepatocyte growth factor (HGF)/epithelial–mesenchymal transition (EMT) signalling pathway is particularly evident in this context, with a significant and positive correlation observed between the expression of PD-L2 and the pathway. Viral infections, such as those caused by human papilloma virus (HPV) and Epstein–Barr virus (EBV), also contribute to the upregulation of PD-L2, which in turn facilitates the development of HNSCC [16]. By comparison, the regulation by super-enhancers is of greater significance. It not only abolishes the inhibitory effect of INF-γ, but also facilitates the formation of PD-L1 and PD-L2 topology-associated domains (TADs), which significantly enhance the effect of super-enhancers, leading to a substantial elevation in PD-L2 expression in HNSCC. In contrast, glycosylation exerts a relatively limited influence on the quantitative expression of PD-L2. The expression ratio of PD-L2 with other B7/CD28 family members, particularly those with higher PD-L2/inducible T-cell costimulatory protein (ICOS) ratios, has been associated with a poorer prognosis.

##### B7-H3

B7 homolog 3 protein (B7-H3), also known as CD276, is a type I transmembrane protein encoded by chromosome 15. It can be presented on the cell surface or in the cytoplasm and can even be secreted extracellularly in a soluble form [19]. B7-H3 exhibits sequence similarity to the extracellular structural domains of other members of the B7 family. However, the identity of the B7-H3 receptor remains elusive. Several potential candidates have been proposed, including TREM-like transcript-2 (TLT-2), IL20Rα and phospholipase A2 receptor 1 (PLA2R1), but the precise mechanisms of action of these receptors are unclear, and the current evidence does not fully elucidate the complex effects of B7-H3 on tumour behaviour and the TME [20,21].

B7-H3 mRNA is expressed in the majority of normal tissues but still significantly lower than the level of expression in HNSCC. This difference facilitates antibody-based immunotherapy as it is highly expressed on differentiated tumour cells with limited heterogeneity, favouring selective killing of cancer cells [22,23]. In certain cases, soluble B7-H3 proteins, have been observed to co-stimulate CD4 and CD8 T cell proliferation and IL-12 production through matrix metalloproteinases (MMPs), thereby facilitating the clearance of tumours [21]. This may be related to the activation of specific regulatory factors (e.g. Bromodomain and Extra-Terminal domain containing-4 (BRD-4), Immunoglobulin-like transcript-4 (ILT-4)), the p38 mitogen-activated protein kinase (MAPK)-eIF4E signalling axis, methylation and the action of different ligands [20,22]. In HNSCC, high expression of B7-H3 is associated with a poorer prognosis and positively correlated with larger tumour size, later clinical stage, lower patient survival and infiltration of M2 macrophages and myeloid-derived suppressor cells (MDSCs) [24]. This includes the reduction of immune surveillance, the formation of an immunosuppressive microenvironment and resistance to some common ICs [23]. Notably, B7-H3 expression was significantly higher in CSCs than other cells in HNSCC, indicating that B7-H3 is also an enriched surface marker for HNSCC CSCs [20]. This may be related to the fact that CSCs utilize B7-H3 to escape from immune system during initiation, progression and metastasis of HNSCC.

##### VISTA

V-type immunoglobulin domain-containing suppressor of T cell activation (VISTA), also known as PD-1H, B7-H5, Dies1, Gi24, DD1α, C10, etc., is an IC molecule harbouring an immunoglobulin structural domain that potently inhibits both *ex vivo* and *in vivo* T cell-mediated immune responses, initially characterized in 2011 [25–27]. VISTA stands out as the most conserved member of the CD28-B7 family, featuring an IgV structural domain that is highly homologous to PD-L1, but functions independently of the PD-1 pathway [28]. Despite its high homology with PD-L1 and other B7 family members, the cytoplasmic tail of VISTA lacks the classical immunoreceptor tyrosine-based inhibitory motif/immunoreceptor tyrosine-based activation motif (ITIM/ITAM) motifs and IgC domains [29,30].

During oncogenesis, VISTA may play a dual role as a ligand for P-selectin glycoprotein ligand-1 (PSGL-1) on T cells and as a receptor for V-set and Ig domain containing-3 (VSIG-3) on tumour or myeloid cells. VISTA selectively binds to PSGL-1 under acidic conditions and to VSIG-3 at physiological pH condition, engaging in bidirectional signalling and modulating the levels of several cytokines through the phosphorylation of multiple molecules (Linker for activation of T cells (LAT), SH2 domain-containing leukocyte protein of 76 kDa (SLP76), Phospholipase C gamma-1 (PLCγ-1), etc.) [14,31]. The role of VISTA in various cancers remains ambiguous. It serves as both an inhibitory IC and potentially a co-stimulatory checkpoint in certain cancer types, such as oesophageal and gastric cancers [30]. Its inhibitory effects are typically predominant in regulating adaptive immune resistance, immune evasion, intrinsic induction and immune tolerance, particularly in MDSCs, where it is upregulated in a hypoxia-inducible factor (HIF)-1-dependent manner compared to peripheral lymph nodes [32,33]. High expressions of VISTA on MDSCs in patients from a broad spectrum of cancers have been correlated to acquired resistance to anti-CTLA-4 and anti-PD-1/PD-L1 therapies, suggesting a compensatory inhibitory role of VISTA in certain malignancies [34].

In HNSCC, particularly oral squamous cell carcinoma (OSCC), there is a significant increase in VISTA protein expression compared to normal oral mucosa. This expression is strongly associated with high expression of other ICs and reduced overall survival (OS) [25,33]. There is a correlation between VISTA protein expression and the expression or activation of IL13Rα2, phosphoinositol-3 kinase (PI3K) and p-STAT3, with these molecules being highly expressed or activated in human OSCC. Notably, overexpression of IL13Rα2 is associated with increased transforming growth factor (TGF)-β1 in cancer, a significant cytokine for tumour metastasis and recruitment of MDSCs. p-STAT3 activation prevents the maturation of myeloid cells, and PD-L1 expression is driven through the STAT3 pathway. The effects of PI3K-AKT signalling pathways, which are independent of those of CTLA-4, may indicate that combined CTLA-4 and VISTA blockade in HNSCC may be more effective than combined PD-1 and VISTA blockade [25,28].

##### BTLA

B and T lymphocyte attenuator (BTLA), an inhibitory receptor of the IgSF, is a type I membrane glycoprotein that plays a pivotal role in the modulation of immune responses. BTLA shares structural similarities with CTLA-4 and PD-1, featuring a single C-type Ig structural domain and three conserved tyrosine motifs within its cytoplasmic. The receptor includes the Growth Factor Receptor-Bound Protein-2 (Grb-2) recognition motif, ITIM and immunoreceptor tyrosine-based switch motif (ITSM) [35–37]. Notably, splice variants and soluble forms of BTLA are also recognized [38]. BTLA interacts with ligands such as herpesvirus entry mediator (HVEM) and its homologues, and this process negatively regulates cellular effects and antagonizes the effects of tumour necrosis factor (TNF) superfamily member 14 (LIGHT)-HVEM axis [38,39]. The binding represents a significant inhibitory pathway in the HVEM-associated regulatory network and challenges the conventional view that receptors only bind to ligands within the same family. It is the only receptor–ligand interaction that directly links the TNF receptor to the IgSF receptors [36,38].

In HNSCC, the pattern of BTLA expression is markedly distinct from that observed in physiological conditions. It is characterized by a persistent increase in BTLA expression on specific lymphocytes (mainly CD4^+^ T cells). Genetic polymorphisms associated with these lymphocytes are linked to increased susceptibility to a range of cancers and poorer prognosis [6,39,40]. Soluble BTLA (sBTLA) and PD-1 expression in OSCC is over four times higher compared to normal tissues, positively correlates with susceptibility to OSCC and significantly negatively correlates with OS and tumour mutation burden (TMB) [41,42]. However, sBTLA does not serve as a prognostic marker for advanced HNSCC that has undergone chemotherapy or immunotherapy in patients with advanced HNSCC, and the underlying mechanism still remains unclear [42].

#### TIM family

T cell immunoglobulin domain and mucin domain-3 (TIM-3), was initially identified in 2001 by researchers studying the role of interferon (IFN)-γ-producing T cells in asthma susceptibility genes [43].

In tumours, TIM-3 has been demonstrated to diminish the adhesion capacity of tumour cells and engage in autocrine regulation through signalling pathways involving its ligand [44]. TIM-3 on cancer cells has been shown to promote EMT and to regulate tumour biological behaviour through the MAPK, NF-κB, SMAD7/SMAD2/SNAIL1 and other signalling pathways [45]. On TAMs, TIM-3 regulates tumour biological behaviour by enriching IL-4, which has anti-inflammatory and pro-tumour effects [44].

The function of TIM-3 depends on the type of ligands. Four ligands for TIM3 have been identified: galactose lectin-9 (Gal-9), high mobility group protein B1 (HMGB1), phosphatidylserine (PtdSer) and carcinoembryonic antigen cell adhesion molecule 1 (CEACAM-1) [43]. Gal-9 amplifies cell death signals and promotes apoptosis through the formation of the (Gal-9/TIM-3)_n_ complex. The formation of a heterodimer between TIM-3 and CEACAM-1 in the cytoplasm or nucleus may be indicative of a poor prognosis.

In HNSCC, TIM-3 exerts a pivotal role, with a substantial proportion of cases (91.3%) displaying TIM-3^+^ tumour-infiltrating lymphocytes (TILs), either intratumourally or in the peritumoural stroma [46]. TIM-3 exerts its influence predominantly in the advanced stages of tumourigenesis within HNSCC. In the early phases (stages I and II), the presence of TIM-3^+^ TILs does not significantly correlate with survival duration, and TIM-3^+^ CD4^+^ T cells are capable of effectively secreting IFN-γ, thereby contributing to antitumour immunity [47]. Nevertheless, in the intermediate and late stages (stages III and IV), there is an expansion of TIM-3^+^ regulatory T cell (Treg) cells, which impede the function of effector T cells. This is frequently accompanied by high CEACAM1 expression [46]. TIM-3 expression is posited to modulate the EMT in tumours, potentially facilitating metastatic spread. This promotion of EMT may be mediated through the upregulation of MMP9 and MMP2, alongside the modulation of the MAD7/SMAD2/SNAIL1 signalling axis, leading to an increase in mesenchymal and a concomitant decrease in epithelial markers [45]. Notably, exosomes derived from nasopharyngeal carcinoma (NPC) cells harbour galectin-9, which, upon circulation in the TME and bloodstream, binds to TIM-3 on Th1 cells, eliciting an immunosuppressive response [45].

#### Other members of IgSF

##### LAG-3

Lymphocyte activation gene-3 (LAG-3), the third IC pathway to be therapeutically harnessed in oncology, was identified as a structural homologue of CD4 in 1990 [48]. This CD4-like inhibitory receptor is characterized by four immunoglobulin-like domains (D1–D4) and shares binding sites with CD4 for major histocompatibility complex (MHC) class II and classical ligands [49]. In the TME, LAG-3 is frequently upregulated and engages with MHC-II via its D1 domain, thereby competitively inhibiting MHC-II–CD4 interactions and disrupting the interaction of the co-receptors CD4 and CD8 with the tyrosine kinase. This results in the attenuation of proximal T cell receptor (TCR) signalling and a subsequent impairment of effector T cell functionality [48–50]. Beyond MHC-II molecules, LAG-3 interacts with a range of potential ligands, including galectin-3 (Gal-3), liver and lymph node sinusoidal endothelial cell C-type lectin (LSECtin), fibrinogen-like protein-1 (FGL-1) and α-synuclein pre-fibril (α-syn PFF).

Pan-cancer analyses have discovered that HNSCC is characterized by elevated LAG-3 expression, which is significantly associated with adverse prognosis and resistance to therapeutics [51]. In HNSCC patients, LAG-3 levels in peripheral blood exhibit a negative correlation with ADAM10, an enzyme that can cleave LAG-3 from the cell surface, implying a tight regulatory control of LAG-3 by ADAM-mediated ectodomain shedding. Within the TME of HNSCC, LAG-3 can modulate TCR signalling via a negatively charged amino acid motif in its cytoplasmic tail or by localizing to the immunological synapse [48]. At the immunological synapse, LAG-3 interaction with MHC class II molecules suppresses T cell activation and proliferation while enhancing the expression of TGF-β1 and IL-10, leading to apoptosis, anergy and cell cycle arrest in activated T cells, which ultimately dampens antitumour immune responses [52]. Furthermore, LAG-3 can suppress the expression of attractants including CXCL1 and CCL2, curtailing the recruitment of immune cells and thereby inhibiting immune responses. Emerging clinical evidence demonstrates that LAG-3 and CTLA-4/PD-1 are not merely redundant checkpoints; instead, they selectively restrict two distinct CD8^+^ TIL subsets – namely, an ‘exhausted-IFN-I-high’ population and an ‘effector-memory-IFN-I-low’ population [53].

##### TIGIT

T cell immunoglobulin and ITIM domain (TIGIT), emerged as a notable IC receptor in 2009, classified in the poliovirus receptor (PVR) family within the broader IgSF and more specifically within the nectin family [54].

Throughout the process of tumourigenesis, TIGIT is predominantly overexpressed on T cells, natural killer (NK) cells, Tregs and certain B cell populations, thereby indirectly modulating antitumour immune responses through its effects on dendritic cells, Tregs and macrophages [54]. In addition, the co-expression of TIGIT alongside other checkpoint molecules, including PD-1, TIM-3 and LAG-3, on TILs is particularly increased in HNSCC, a phenomenon associated with T cell exhaustion [55,56].

As an IC receptor within the IgSF, TIGIT interacts with ligands such as CD155 (PVR or Nectin-5), CD113 (PVRL3 or Nectin-3), CD112 (PVRL2 or Nectin-2) and PVRL4 (Nectin-4) to suppress T cell activity, facilitating tumour evasion of immune surveillance [54]. Highly expressed on diverse tumour cells and tumour-associated myeloid cells, TIGIT engages with its counterpart on CD8^+^ T cells and NK cells, providing inhibitory signals that suppress antitumour immunity [57]. The interaction between TIGIT and CD155 is frequently orchestrated by MDSCs [8,58]. Upon ligation with CD155, its major inhibitory receptor, TIGIT induces the formation of dense nanoscale clusters on the T cell surface [57]. This engagement potentially disrupts signalling cascades including MAPK, NF-κB and PI3K, limiting cytokine release, impeding glycolysis, reducing IFN-γ production, promoting IL-10 secretion from dendritic cells and enhancing the proliferation of FOXP3^+^ Treg cells, culminating in T cell and NK cell exhaustion [54,57,59,60].

TIGIT exhibits a comparable expression pattern to PD-1 on T lymphocytes, yet its impact on the signalling pathway occurs at an earlier stage [54,58]. The co-expression of these two markers on MDSCs is both frequent and mechanistically intertwined, but each employs unique mechanisms to limit the binding activity of CD226, providing a rational basis for the combined targeting of PD-(L)1 and TIGIT in cancer immunotherapy [61].

Increased expression of TIGIT in a variety of tumours, including melanoma, acute myeloid leukaemia, oesophageal SCC, neuroblastoma and HNSCC, is closely associated with tumour progression [56,59,62]. Research indicates that TIGIT cooperates with HIF1-α to enhance tumour cell invasiveness, colony formation and angiogenesis, and subsequently promotes tumour development and progression [54].

In the TME of HNSCC patients, TIGIT expression increases over time, in contrast to its decrease in peripheral blood mononuclear cells, and is often co-expressed with other IC molecules. In contrast, the increased sensitivity of TIGIT results in increased expression without the upregulation of other IC molecules in OSCC [8,9,56,59]. Overexpression of TIGIT in cancer cells or tumour-infiltrating stromal cells typically predicts poorer OS and is linked to pathological grading and lymph node metastasis [8]. In OSCC, patients with higher levels of TIGIT expression paradoxically have a better prognosis, suggesting a controversial clinical role for TIGIT [60].

### A member of TNFR family – GITR

The majority of IC molecules belong to the IgSF, which typically have inhibitory immunological effects when activated. In contrast, the TNFR family often induces activating immunological effects when activated, which has attracted increasing attention and prompted extensive research on TNF family agonists. To date, extensive research has been carried out on key members of the TNFR family, including OX40 and 4-1BB, while emerging costimulatory IC molecules have recently gained significant attention and triggered a wave of research enthusiasm in the field of immunotherapy. Among the emerging costimulatory IC molecules, glucocorticoid-induced TNFR-related protein (GITR) stands out as the most significant IC molecule that has entered clinical trial stage.

GITR is a type I transmembrane glycoprotein within the TNFR family, predominantly found on T cells and NK cells. Its expression is observed in a variety of tissues and cell types, including Tregs, activated T cells, NK cells, polymorphonuclear cells and certain non-immune cells like osteoclasts, with particular prominence on CD4^+^CD25^+^ Tregs [63,64]. Activation of GITR enhances the functionality of effector T cells (Teffs) while suppressing Tregs, thereby increasing the immune system’s ability to target and eliminate tumour cells [65].

The ligand for GITR, widely recognized as GITRL (also known as TNFSF18), is expressed on myeloid cells and is characterized as a type II transmembrane protein with a canonical TNF homology domain (THD). GITRL is sparsely expressed on APCs but is upregulated during activation [66,67]. Preclinical studies confirm that GITR activation increases the activity of CD8^+^ and CD4^+^ effector T cells and decreases tumour-infiltrating Tregs, particularly in HNSCC. Another ligand for GITR, secreted and transmembrane protein 1 A (SECTM1A), can be expressed as both a transmembrane and secreted protein, but its function remains elusive [64].

In HNSCC, elevated GITR expression correlates with a favourable prognosis, which may be related to the inhibitory effects of tumour cells and its prevalence [64,68]. On the one hand, the GITR pathway can enhance the antitumour efficacy of T cells by upregulating IL-2, IL-2 receptor α and IFN-γ, thereby enhancing the proliferation and effector functions of CD4^+^ and CD8^+^ T cells [67]. On the other hand, GITR activation attenuates FOXP3 expression in Treg cells by inhibiting Helios-dependent STAT5 activation, thereby promoting Treg cell instability [69].

## Emerging therapies of novel ICs

These novel therapeutic targets have paved way for overcoming the immunosuppressive TME of tumours, offering a promising avenue to emerging therapies. So far, these nine ICs have been studied to varying degrees (Tables 1 and 2).

**Table 1.** Preclinical experiments and clinical trials performed based on novel ICIs in the article.

Target	Drugs/measures	Cell lines/ models/cohorts	Function/mechanism	Influence	Focus on primary/metastatic tumour or both	Ref
PD-L2	ODN TTAGGG, Chloroquine	SCC154, SCC099	Interfered with CpG ODN colocalization with TLR9 in endosomes.	Abrogated the up-regulation of both PD-L1 and PD-L2.	Primary	71
	Fludarabin, cryptotanshinone	HSC-2	Suppressed the phosphorylation of STAT1/3.	Decreased the relative number of PD-L2-positive cells.	Primary	72
	Tocilizumab	SCC15 and SCC-25	Blocked IL-6, decreased PD-L2 mRNA and protein levels.	Prolonged OS.	Primary	19
	Curcumin	SCC15 and FaDu	Decreased the expression of suppressive IC receptors and their ligands (PD-L1, PD-L2 and Galectin-9) in the TME.	Effectively restored the ability of CD8+ cytotoxic T cells.	Primary	73
B7-H3	MJ18	Tgfbr1/Pten 2cKO	Directly blocked B7-H3.	Reduced the tumour burden of head and neck.	Primary	74
	MGC018	PDX	Was internalized by tumour cells and damage their DNA.	Exhibited cytotoxicity toward B7-H3-positive tumour cells, and exhibited bystander killing effects.	Metastatic	75
	JQ1、iBET-151	SCC1, SCC22B, SCC23, SCC1R, HN6	Disrupted super enhancers and inhibited expression of CD276 at the mRNA and protein levels.	Significantly decreased overall lesion numbers and lesion areas.	Primary	76
	sh-RNA	HOEC, SCC-9, SCC-15, CAL-27, SCC-25	Inhibited the expression of B7–H3 and enhanced the killing effect of CD8 + T cells.	Inhibited HNSCC cell proliferation, migration and invasion *in vitro*.	Primary	77
	Enoblituzumab	Checkpoint inhibitors-naïve HNSCC patients	Increased IFN-γ expression and up-regulated PD-L1 on NK cells.	Had an acceptable safety profile and robust activity in patients with HNSCC.	Primary	78
VISTA	MIH63	SCCVII	Converted CD8+ T cells into functional effector T cells.	Inhibited tumour growth regression and efficiently induced of CD8+ T cell activation.	Primary	30
	Knockdown of CMTM6	SCC7	Unknown	Showed a significant decrease in the expression of VISTA in T cells.	Primary	79
TIM-3	RMT3-23	Tgfbr1/Pten 2cKO	Directly blocked TIM-3.	Restored effector T-cell function by targeting CD4 + TIM3+ cells and CD8 + TIM3+ cells and decreasing MDSCs.	Metastatic	80
	Anti-TIM-3 antibody	MOC2 and LY2 cell lines	Enhanced T-cell cytotoxicity, decreased Tregs.	The response was not durable, and analysis of relapsed tumours revealed resurgence of Tregs.	Primary	81
	Clone 2E2 from Biolegend	TILs from HNSCC patients	Downregulated Tim-3 expression after PD-1 blockade *ex vivo* in mouse TIL.	Reversed the suppressive function of HNSCC TIL Tregs.	Primary	82
	Anti-TIM-3 monoclonal antibody	SCC-7, SCC090	Prevented the combination of Galectin-9/TIM-3.	Enhanced the efficacy of HNSCC immunotherapy, particularly in the case of HPV16+ HNSCC.	Primary	83
LAG-3	Anti-LAG-3 monoclonal antibody	B16-F10 and B16-gp100 cells	Reduced the levels of CCL2 and CXCL1.	Reshaped antitumour response in HNSCC.	Primary	84
	CRISPR-Cas9	MOC1AhR KO	Knocked out AhR.	Significantly decreased Tumour growth after AhR deletion.	Primary	85
	HPV E6/E7 antigen stimulation	C-225 and C100	May related to TME differences.	C-225 elicited complete eradication, whereas C-100 grew progressively.	Primary	52
TIGIT	Motolimod	HPV- HNC cell line JHU029	Activated monocytes, DC, and NK cells through NF-kB–mediated signalling.	Decreased induction of Treg and reduced markers of suppression, including TIGIT.	Primary	86
	TIGIT mAb	Tgfbr1/Pten 2cKO	Decreased the inhibitory function of Tregs and the mRNA expression level of Arg-1 in MDSCs.	Tumour progression was significantly delayed in mice treated with TIGIT mAb.	Metastatic	9
	Anti-human TIGIT monoclonal antibody	Human PBMC and TILs	Barely detectable on both CD4+ and CD8+ T cells after TIGIT blockade in vitro.	Restore the function of dysfunctional CD4+ and CD8+ T cells.	Both	60
	TIGIT mAb	Tgfbr1/Pten 2cKO mice	Increased the expression level of the activation receptor CD226 on CD8+ T cells.	Effectively inhibited the growth of HNSCC.	Primary	10
	Anti-TIGIT antibodies and AZD6738	MOC2 SCC7	Enhanced radiotherapy-induced inflammation in the TME.	Improved ATRi/RT treatment in terms of both tumour growth and tumour-specific survival.	Primary	87
	TIGIT mAb	a TMA cohort of OSCC	Unknown.	Inhibited tumour growth.	Primary	61
GITR	MEDI1873, AMG 228, BMS-986156, TRX518, MK-4166	HNSCC cells	Activated GITR.	Demonstrated a good safety profile and potential activity.	Metastatic	56
	MK-4166	Solid Tumour cell lines (HNSCC included)	Activated GITR.	Decreased GITR availability on circulating T cells with increasing doses.	Primary	68

### Therapies of emerging ICIs

#### PD-L2 therapy

In HNSCC, predominant therapies target upstream signalling pathways, including STAT3/FUT8, IL6-IL6R and TLR9. The incorporation of polyclonal antibodies and combination chemotherapy strategies is also deemed highly significant.

Notably, STAT3/FUT8-mediated PD-L2 glycosylation is a pivotal regulator of immune evasion and responsiveness to anti-epidermal growth factor receptor (EGFR) therapies in HNSCC, indicating PD-L2 glycosylation may be a potential strategy to enhance the therapeutic response of HNSCC patients to anti-EGFR therapies [70]. Within the TLR9 pathway, chloroquine inhibits TLR9 activation by obstructing endosomal acidification and the interaction between DNA and TLR9, whereas ODN TTAGGG interrupts TLR9 activation by disrupting the co-localization of CpG oligodeoxynucleotides, thereby preventing the upregulation of PD-L2 [71]. Curcumin in HNSCC can simultaneously suppress multiple ICs, including PD-L2, demonstrating the ability to reduce the intrinsic expression of PD-L2 independently of immune cells and to inhibit EMT, potentially exceeding the efficacy of monoclonal antibodies targeting a single IC [72].

Within the context of cisplatin chemotherapy of HNSCC, the upregulation of PD-L2 in response to cisplatin chemotherapy is a critical factor that must not be disregarded. Importantly, STAT1 inhibitors, including fludarabine, and STAT3 inhibitors, such as andrographolide, have been shown to partially attenuate cisplatin-induced PD-L2 expression, resulting in a decreased proportion of PD-L2^+^ cells compared to cisplatin alone [73]. In the neoadjuvant setting for HNSCC, Zhang et al. demonstrated – using both TCGA data and an institutional cohort of 132 patients receiving pre-operative chemotherapy or chemoradiation – that high PD-L2 expression constitutes an independent prognosticator of poor outcome, increasing the 2-year distant-metastasis hazard by 2.3-fold (HR 2.31, 95% CI 1.25–4.28) [18]. Complementarily, the ongoing phase II/III KEYNOTE-689 trial (NCT03765918) has pre-specified PD-L2 as a stratification biomarker; interim data show that patients with PD-L2 ≥ 1% achieve a pathological complete response rate of only 18.9% after pembrolizumab plus chemotherapy, significantly lower than the 39.4% observed in the PD-L2 < 1% subset [74]. Collectively, these findings position PD-L2 as a discriminatory marker for suboptimal response to neoadjuvant immunotherapy and provide a mechanistic rationale for perioperative co-targeting of the IL-6/JAK pathway.

In the realm of future clinical practice, it is essential to acknowledge that PD-L2, despite its lower expression compared to PD-L1, is detectable and warrants consideration when designing treatment strategies with PD-1 ICIs. Furthermore, an array of small molecule drugs, monoclonal antibodies and PD-L2-targeted vaccines are under intensive investigation [17].

#### B7-H3 therapy

In HNSCC, strategies targeting B7-H3 primarily include monoclonal antibody-drug conjugates (ADCs) and small molecule inhibitors. These therapeutics interact directly with B7-H3 through specific binding and indirectly by inhibiting epigenetic modifications of B7-H3.

MJ18, a monoclonal antibody against B7-H3, has been shown to significantly reduce tumour volume in the Tgfbr1/Pten 2Cko mouse model without causing additional cytotoxicity and to effectively reverse the immunosuppressive state [75]. Enoblituzumab, another monoclonal antibody targeting B7-H3, enhances antibody-dependent cellular cytotoxicity (ADCC) through the engagement of the Fcγ receptor, meriting further investigation [76]. In HNSCC, the ADC MGC018 was particularly effective, reducing tumour volume by 98% and exhibiting a ‘bystander effect’, eliminating both B7-H3-expressing and adjacent non-B7-H3-expressing tumour cells [77].

In the area of epigenetics, the small molecule inhibitors JQ1 and Ibet-151 have demonstrated the ability to inhibit the activity of BET (Bromodomain and Extra Terminal domain) proteins, thereby impeding the function of the B7H3 super-enhancer and dampening the expression levels of B7H3, as well as inhibiting the proliferation of HNSCC [78]. The utility of short hairpin RNA (shRNA) technology also deserves attention as it has the potential to disrupt the sponge-like activity of LINC01123, thereby preventing the upregulation of B7-H3 expression by miR-214-3p [79]. However, to date, no shRNA-based therapeutics have advanced to the clinical trial stage. In neoadjuvant therapy of resectable HNSCC, high tumoural B7-H3 (CD276) expression is an independent predictor of post-operative distant metastasis: in a tissue-microarray study of 289 patients undergoing curative-intent surgery, those with ≥30% of tumour cells exhibiting strong (3+) B7-H3 staining had a 3-year distant-metastasis rate of 46% versus 18% in the negative/low cohort [80]. Although no peri-operative trials of B7-H3 blockade have yet been reported, early-phase data from recurrent/metastatic disease provide proof-of-concept: in a multicentre phase I/II trial, 18 PD-L1-negative, platinum-refractory HNSCC patients treated with the Fc-optimized B7-H3 monoclonal antibody enoblituzumab (MGA271) plus pembrolizumab achieved an objective response rate of 33% and a disease-control rate of 72%, with grade ≥3 immune-related adverse events in 11% [76].

Combination therapies represent a promising therapeutic strategy. Despite substantial evidence supporting the oncogenic effects of B7-H3, there are currently no FDA-approved drugs targeting B7-H3. The elusive status of B7-H3 receptors makes this molecule a challenging target for pharmacological intervention [22].

#### VISTA therapy

VISTA holds therapeutic promise across a spectrum of cancers, and the development of numerous VISTA-targeting antibodies represents significant progress.

Among these, CA-170 and JNJ-61610588 are under extensive investigation. CA-170, the first licensed small molecule inhibitor of VISTA/PD-L1, selectively targets these targets. Its oral bioavailability mitigates infusion-related risks and it exhibits potential efficacy in OSCC [26]. JNJ-61610588, a humanized anti-VISTA monoclonal antibody, exerts its primary effects in conjunction with PD-L1/PD-L2, markedly improving the efficacy of IC blockade [30]. In certain solid tumours, the binding of VISTA.4, VISTA.5 and VISTA.18 to VISTA is FcR-dependent and more vulnerable to the acidic TME, potentially reducing therapeutic efficacy, which might be effective in HNSCC. HMBD-002, an antibody independent of FcR, specifically targets the C-C’ loop region of VISTA, a critical site for interaction with potential ligands such as VSIG3. HMBD-002 has shown good safety profiles, though its application remains limited [34]. VISTA is markedly up-regulated on myeloid cells following neoadjuvant radiotherapy in HNSCC and OSCC, driving CD8^+^ T-cell exhaustion and independently predicting local relapse and distant metastasis. Preclinical models demonstrate that VISTA blockade reprograms M2-TAM and PMN-MDSC and synergizes with PD-1 inhibition to amplify the abscopal effect of radiation. Humanized antibodies HMBD-002 and CI-8993 have established safety profiles, providing a rationale for neoadjuvant combination trials enriched by VISTA^+^CD11b^+^ ≥30% [81].

Despite the distinctive expression profile of VISTA in HNSCC, direct investigations of the role of VISTA in HNSCC are sparse. Clinical trials (NCT04475523, NCT02812875) have been discontinued without reported results [30]. Nevertheless, VISTA remains a promising target for IC therapy and may play a pivotal role in future HNSCC treatment strategies.

#### BTLA therapy

The role of BTLA is intricately complex. Augmenting of BTLA/HVEM signalling may offer therapeutic benefits in autoimmune diseases, while inhibition of this pathway could potentially enhance immune responses to tumours or pathogens. In oncology, the suppressive and prognostic roles of BTLA and HVEM in certain solid tumours have been strongly supported by a number of studies. However, data concerning the anticancer efficacy of targeting the BTLA/HVEM axis in HNSCC remain scarce [6,39].

In haematology, the modulation of CD19 and the subsequent activation of the BTLA/HVEM axis has slowed the progression of Hodgkin’s lymphoma in murine models and mitigated the systemic immunosuppression associated with CAR-T therapy [39]. Clinical trials NCT04477772 and NCT05000684 have demonstrated that the BTLA monoclonal antibody tifcemalimab, both as a monotherapy and in combination with a PD-L1 antibody, effectively inhibited lymphoma and extensive-stage small cell lung cancer with a favourable safety profile [42]. The BTLA monoclonal antibody TAB004 has induced an upregulation of CD8^+^ T cells, resulting in partial remission in 1 out of 19 melanoma patients and disease stabilization in 6 patients (NCT04137900).

It is unfortunate that there are very few clinical studies that reported on BTLA in HNSCC, possibly due to the significantly higher expression of Slag-3 relative to BTLA in HNSCC, which may overshadow the biological impact of BTLA [82]. To date, only one study of BTLA antibodies in HNSCC has been identified (NCT04929080), with results pending, leaving the therapeutic efficacy in HNSCC to be further established. Future research should focus on conducting neoadjuvant clinical trials targeting BTLA, exploring its potential as a diagnostic biomarker and evaluating the efficacy and safety of its combination with anti-PD − 1/PD-L1 therapies, thereby providing more effective treatment strategies for OSCC patients.

#### TIM-3 therapy

TIM-3 is a relatively well studied novel IC molecule that promotes T cell dysfunction and exhaustion in cancer and chronic viral infections [83]. It has shown promise as a potential therapeutic target in HNSCC.

In HNSCC, monotherapy targeting TIM-3 has demonstrated modest efficacy in the gfbr1/Pten 2cKO mouse model, significantly reducing tumour volume and increasing CD4^+^ and CD8^+^ T cell populations within the TME and periphery, while also decreasing MDSC counts [84]. However, TIM-3 is more commonly used in combination therapies. In HNSCC, TIM-3 expression alone does not necessarily indicate T cell exhaustion; rather, a secondary increase in TIM-3 following certain treatments, such as radiotherapy, chemotherapy or anti-PD-1 therapy, may suggest T cell exhaustion [85]. Oweida et al. discovered that the addition of anti-TIM-3 to radiotherapy and anti-PD-L1 treatment significantly increased the number of functional effector T cells (CD44^+^IFNγ^+^), leading to a substantial prolongation of survival [86]. Similarly, Ge et al. observed that the co-administration of anti-PD-1 and anti-TIM-3 treatment significantly enhanced the expression of CD8^+^ T cell cytotoxic cytokines, particularly in HNSCC patients with high circE7 expression [87]. In another mouse HNSCC model, anti-PD-1 treatment downregulated TIM-3 expression on Treg cells and reversed the suppressive function of Tregs, suggesting that these contradictory results may be related to the use of different cell lines and require further exploration [88]. Beyond these findings, the clinical translational value of TIM-3 has been widely recognized, with only NCT0365207 and NCT03058289 having been completed to date. Nevertheless, numerous HNSCC-related clinical trials are ongoing, indicating a promising future for TIM-3. Antibody engineering has delivered PD-1, CTLA-4 and LAG-3 blockade to the clinic; TIM-3 – an evolutionarily related B7-superfamily member – now emerges as the next druggable checkpoint [17]. Its co-expression with PD-1 on exhausted T cells, coupled with a favourable toxicity profile, positions TIM-3 blockade for neoadjuvant exploitation in HNSCC. Mechanistically, anti-TIM-3 mAbs (i) disrupt Galectin-9/PtdSer signalling to relieve dendritic-cell paralysis and potentiate IL-12-driven T-cell responses; (ii) leverage Fc-mediated ADCC/ADCP to eradicate TIM-3+ tumour cells and (iii) spare T-cell intrinsic inhibition, predicting reduced collateral toxicity. Accordingly, Novartis’ sabatolimab is under early-phase evaluation in combination with radiotherapy and PD-1 blockade, offering a low-toxicity, high-efficacy neoadjuvant strategy for locally advanced HNSCC [89,90]. MBG453, is also a selective TIM-3 monoclonal antibody. However, its pharmacotherapeutic impact in HNSCC remains uncharacterized. Investigating and enhancing MBG453′s efficacy in this context may represent a critical strategic approach for TIM-3 checkpoint inhibition in HNSCC.

#### LAG-3 therapy

Currently, the vast majority of therapies targeting LAG-3 and its ligand FGL1 are administered in combination with PD-1 therapies, and the use of LAG-3 antibodies as monotherapy is extremely rare.

In the context of HNSCC, anti-FGL1 therapy can be synergized with anti-PD-1/PD-L1 regimens [91]. Ablation of AhR (as seen in MOC1AhR KO cells) results in attenuated tumour growth within 1 week, leading to complete rejection within 2 weeks, along with an augmentation of activated T cells in tumour-draining lymph nodes (tdLNs) and enhanced T cell signalling within the tumour [92]. Interestingly, the expression levels of LAG-3 fluctuate under the influence of different antigens of the same HPV at different sites, affecting the growth dynamics of HNSCC. Transplantation of HPV E6 antigen expressed in the C-225 cell line and E7 antigen expressed in the C-100 cell line into murine models reveals that HNSCC in C-225 is almost completely eradicated, in contrast to C-100 [50]. Further investigation is warranted to elucidate the underlying mechanisms, which may be related to the different cellular composition within the TME.

While significant progress has been made in targeting LAG-3, current LAG-3 focused oncology treatments primarily aimed to inhibit the interaction between LAG3 and MHC-II molecules. Evidence suggests that LAG-3 blockade may enhance effector functions but potentially at the cost of the antitumour T cell persistence [93]. Moreover, advances have been made in LAG-3-related tracer technologies and antibodies against sLAG-3 in HNSCC, but significant safety concerns remain [51]. Therefore, a deeper understanding of LAG-3′s mechanisms and clinical applications is imperative. In terms of neoadjuvant therapy, LAG-3 and the PD-1/PD-L1 axis constitute synergistic inhibitory circuits; their concurrent blockade complements radiotherapy-induced antigen release, thereby amplifying the *in-situ* vaccination effect of neoadjuvant immunotherapy [94]. An ongoing phase I/II trial (NCT04641871) is prospectively evaluating a neoadjuvant regimen combining LAG-3 inhibition with PD-1 blockade. After definitive chemoradiotherapy, a significant expansion of LAG-3^+^ CD8^+^ and LAG-3^+^ CD4^+^ T cells in peripheral blood mononuclear cells has been observed, implying that radiotherapy can trigger LAG-3-mediated negative feedback. We therefore hypothesize that, within a neoadjuvant chemoradiotherapy plus LAG-3 blockade protocol, a postoperative decline in circulating LAG-3^+^ T cells would signify reversal of immunosuppression and may serve as an early pharmacodynamic biomarker of efficacy [17]. In NCT04080804, patients who received nivolumab plus relatlimab (Nivo + Rela) and achieved a major pathologic response (pTR-2) exhibited a marked contraction of the exhausted subset after just 4 weeks of therapy, indicating that LAG-3 inhibition can reverse T-cell exhaustion prior to surgery. These findings position LAG-3 as a potential cornerstone molecule for first-line neoadjuvant therapy in HNSCC [53].

#### TIGIT therapy

T cells express TIGIT in a pattern analogous to PD-1 on T lymphocytes, rendering it a promising therapeutic target [58].

In the field of HNSCC, TIGIT is emerging as a prominent therapeutic target. Studies indicate that in murine models, TIGIT mAb treatment results in the inhibition of the CD155/TIGIT axis and an upregulation of CD226 levels, with a concomitant decrease in the frequency of Tregs infiltrating peripheral immune organs, local circulation and the TME, whereas TIGIT expression is augmented following PD-L1, radiotherapy and ATR (Ataxia Telangiectasia and Rad3-Related) inhibitor treatments [95]. In comparison, TIGIT mAb-treated mice exhibit higher frequencies of tumour-infiltrating CD8^+^ T cells expressing IL2/TNF-α/IFN-γ and CD4^+^ T cells expressing IL2, without altering the ratio of memory CD4^+^ T cells [8,9]. In addition, the small molecule Hemin can bind to TIGIT, obstructing its interaction with PVR while inducing ferroptosis, offering novel therapeutic insights for HNSCC [58]. Non-TIGIT-targeting therapies, such as the anti-CTLA-4 antibody Cetuximab and the TLR8 agonist Motolimod, may also downregulate TIGIT expression in HNSCC [96]. Notably, in OSCC *in vitro* experiments, the capacity of CD4^+^ and CD8^+^ T cells to produce IL-2, IFN-γ and TNF-α is markedly enhanced following 3 days of stimulation with anti-CD3/CD28 and anti-TIGIT mAb [59]. The engagement of FcγR is considered a critical factor in the development of anti-TIGIT antibodies; however, it primarily impacts CD96 activity, and since TIGIT shares ligands with CD96, the interplay between CD96 and TIGIT represents a significant avenue for future investigation [97]. In neoadjuvant therapy, especially in motolimod–cetuximab combination therapy, findings have demonstrated that pathological responders exhibit sustained baseline co - expression of PD - 1 and TIGIT on peripheral CD8 + T - cell subsets [96]. These observations mechanistically align with a recent single-arm trial in locally advanced HNSCC, wherein TIGIT geometric mean fluorescence intensity positively correlates with terminally exhausted T cell infiltration [98]. Collectively, this tripartite evidence establishes TIGIT’s role as an ‘immune sentinel’ in HNSCC neoadjuvant therapy, providing early detection of adaptive resistance dynamics and treatment-responsive exhaustion patterns [99].

#### GITR therapy

Beyond inhibiting the aforementioned co-inhibitory pathways, the activation of co-stimulatory pathways to bolster antitumour immune responses is an emerging therapeutic strategy [64]. GITR stands out as one of the most pivotal targets for co-stimulatory pathways stimulation.

In HNSCC, GITR antibodies application is still in a nascent phase. Presently, GITR-directed therapeutics on HNSCC are predominantly at the *in vitro* experimental and preclinical trial stages, with a comparatively scarce number of *in vivo* studies. Among various GITR agonists tested *in vitro*, MEDI-1873 is characterized by its ability to reduce GITR^+^/FOXP3^+^ T cells by over 25%, with a shorter half-life and increased agonistic efficacy [55]. In preclinical *in vivo* trials, antibodies such as AMG228, BMS-986156 and TRX-518 have shown limitations, including transient efficacy. AMG228, in particular, displayed no antitumour activity when administered alone, coupled with poor safety profiles and a range of immune-related adverse events. These issues may be attributed to discrepancies between different mouse models [63,68]. Additionally, while the antibody MK-4166 demonstrates promising efficacy – achieving 90% GITR engagement at a serum concentration of 0.217 µg/mL – it is not without significant adverse reactions, including unconfirmed partial responses in patients [67]. In *in vivo* experiments, existing studies mainly focus on the specific distribution of GITR in immune cells within the TME and the rationale for combined therapy with other ICIs such as PD-1, lacking direct clinical trials to prove whether GITR has a good inhibitory effect on HNSCC [100]. The humanized anti-GITR IgG1 monoclonal antibody GWN323 and the drug molecule HERA-GITRL, which induces optimal trimer assembly of the GITR receptor, as well as a range of other antibodies, require further investigation of their mechanisms of action and therapeutic efficacy [65]. In neoadjuvant settings, GITR constitutes an established biomarker in ovarian cancer management, but remains clinically unexplored in HNSCC neoadjuvant settings [101]. In HNSCC neoadjuvant interventions, OX40 agonism demonstrates capacity to expand CD103^+^CD39^+^ tumour-antigen-specific CD8^+^ TILs, albeit with a response incidence of merely 25% [102]. Crucially, our prior mechanistic analyses reveal fundamental distinctions between GITR and OX40 signalling pathways. Concurrent co-stimulation of both targets may induce dual abrogation of Treg-mediated immunosuppression while synergistically activating CD8^+^ T cells, thereby establishing potential therapeutic synergy with OX40-targeted regimens.

### Integration of novel therapies with ICIs

**Figure 3.**
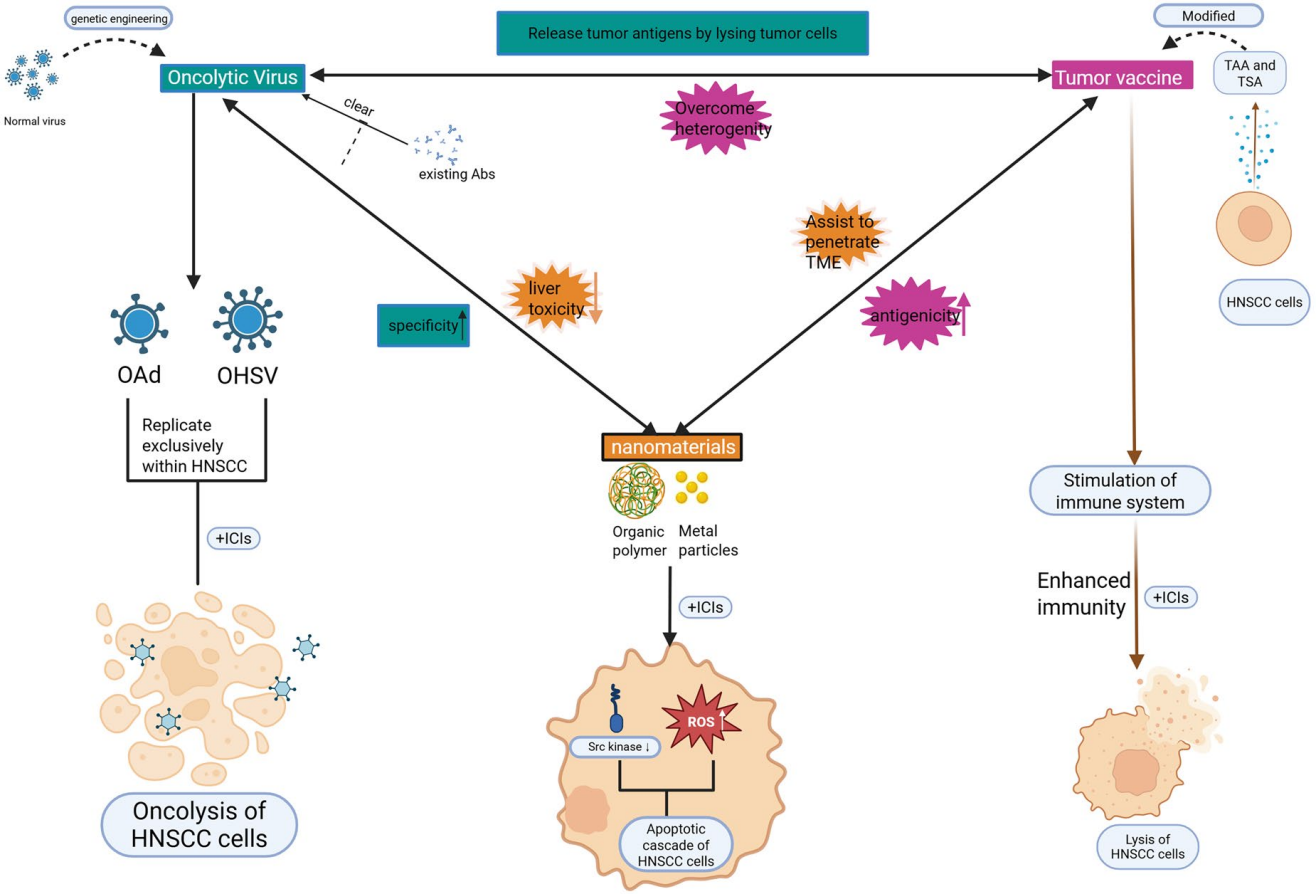
The figure summarizes the sources, determinants, mechanisms and synergistic benefits of cancer vaccines, oncolytic viruses and nanomedicine when combined with novel ICIs. Originally created by BioRender (https://www. BioRender. com).

Although the application of ICIs has shed light on immune therapy for cancers, the exclusive use of ICIs has also been shown to be correlated with a multitude of immune-related adverse events. To improve the delivery efficacy, therapeutic effectiveness and immunological potency of ICIs, they are frequently integrated with alternative therapeutic approaches. The combination of ICIs with conventional treatments, including radiotherapy and chemotherapy, is well established in clinical settings [103]. However, a growing body of clinical research suggests that these combinations with traditional therapies are not devoid of significant adverse effects. Therefore, the convergence of ICIs with innovative therapeutic strategies has received considerable attention in recent years (Figure 3).

#### Combined with nanomaterials

Nanomedicine, an interdisciplinary field that brings together various research domains, concentrates on the nanoscale range of 1 to 1000 nm. Its application in healthcare spans across diagnostics, therapeutics and prevention. Nanocarriers, a category of versatile materials, act as drug vectors, diagnostic agents and theranostic agents capable of integrating diagnostic and therapeutic capabilities. These carriers can be loaded with a variety of therapeutic agents on their surfaces – including fluorophores, small molecules, ICIs and more – to perform diverse functions [104]. Despite their multifaceted capabilities, nanomaterials collectively offer benefits such as enhancing drug properties, targeted drug delivery, crossing biological barriers, enhancing antitumour effects and augmenting immunotherapeutic strategies [105–107].

In HNSCC, synergistic therapy involving nanomaterials and ICIs is beginning to demonstrate its benefits. This efficacy may be due to the nanomaterial’s capacity to optimize the biodistribution, biocompatibility and bioavailability of metabolic modulators, thereby minimizing systemic toxicity and effectively converting an immunologically ‘cold’ TME into a more ‘hot’ and immunologically active state [106]. Nanomedicine has the potential to tackle one of the primary causes of traditional HNSCC treatment failure – the inadequate ability of ICIs to infiltrate target solid tumour tissues – by significantly enhancing the potency of ICIs and facilitating their passage across biological barriers such as the blood-brain barrier [104,108]. Concomitant application with CTLA or PD-1 therapies not only impedes HNSCC progression and mitigates autoimmune-like manifestations but also enhances the cytotoxicity against CAFs and fosters dendritic cell maturation following splenic uptake [105,107]. Zhang et al. engineered an nanoparticle (NP) delivery system (NH2-MSNs) designed for the targeted delivery of small interfering RNA (siS15) against Siglec-15, augmenting the impact of immunotherapy by inhibiting the kynurenine (Kyn)/Siglec-15 axis. Notably, the NP-siS15 group markedly enhanced CD8^+^ T cell infiltration compared to the anti-PD-1 group [109]. Paclitaxel can also be encapsulated in alternative polymeric nanomaterials, such as Abraxane (albumin-bound paclitaxel) and pH-sensitive materials, which have demonstrated efficacy in recurrent/metastatic HNSCC by markedly inhibiting the PD-1/PD-L1 IC and decreasing TIM3^+^ CD8^+^ CTLs [105,107]. In recent years, NBTXR3 has emerged as a pivotal nanoradiotherapy enhancer capable of enhancing energy deposition under ionizing radiation, synergistically potentiating the effects of ICIs [110]. The deployment of a combinatorial regimen involving NBTXR3, XRT and anti-PD1 has been shown to markedly upregulate LAG3 and TIGIT expression, with the subsequent introduction of αLAG3 or αTIGIT further enhancing the control over both primary and metastatic tumours. In contrast, treatment regimens devoid of NBTXR3, even with triple ICIs therapy, have failed to demonstrate robust therapeutic outcomes [110].

Nanomaterials, while safer and more advantageous than traditional therapies, have been subject to limited safety testing, much of which is incomplete [111]. The cost of research is another significant consideration; the intricate preparation process of NPs enhances therapeutic efficacy but hinders large-scale manufacturing and commercial viability. There is a necessity for researchers to develop simpler, more efficient NP preparation techniques to realize their full translational potential and facilitate broad application [112,113]. Advances in material performance are also critical; new methods are needed to control drug delivery to the TME, thereby increasing tumour perfusion, elevating vascular permeability within tumours, and reconfiguring the extracellular matrix. These advances aim to increase the delivery efficiency of NPs and minimize cytotoxic effects, such as those associated with gold nanomaterials [108]. The synergistic action of nanomaterials with other therapeutic modalities and the orchestration of their components to yield beneficial outcomes warrant further exploration [110]. Investigating metabolic interactions among diverse cell types within heterogeneous TMEs, reducing the non-specific clearance of nanomedicines by the reticuloendothelial system, and overcoming the physical and chemical impediments of the ECM to the intratumoural infiltration of nanomedicines and immune cells are important avenues of research [106].

#### Combined with OVs

OVs are a category of live biotherapeutic products capable of selectively targeting cancer cells [114]. The predominant viral entities in oncology therapy are genetically engineered OVs. These OVs are engineered to eliminate genes essential for replication within normal cells, allowing replication only in the tumour cells. This strategy induces tumour cell lysis through virus-mediated oncolysis and replicates within the TME to infect and lyse tumour cells, sparing healthy cells and tissues [115]. Collectively, OVs inhibit tumour progression by directly lysing tumour cells and inducing a systemic anti-tumour immune response.

In HNSCC, five OV products (recombinant human type 5 adenovirus Ankory R©, talimogene laherparep Teserpaturev (G47Δ, DS-1647), talimogene laherparepvec (T-VEC), nadofaragene firadovec-vncg and H101) have been approved for clinical therapy [116]. As an adenovirus, H101 exhibits superior antitumour efficacy in OSCC with elevated Small Nucleolar RNA Host Gene-1 (SNHG1) expression [117]. Other OVs, including T-VEC, have demonstrated the ability to reduce tumour burden in head and neck cancer patients, with enhanced efficacy when combined with ICIs such as pembrolizumab [118]. The potential synergy of TIGIT antibodies with OVs is also significant. The OVs vaccinia virus (VV), engineered to express a single-chain variable fragment (scFv) against TIGIT, known as VV-scFv-TIGIT, markedly transforms the suppressive TME in certain malignancies from a ‘cold’ to a ‘hot’ state [119]. However, excessive TIGIT inhibition may lead to multiple sclerosis, underscoring the need to determine the optimal dosing for TIGIT inhibition as a critical research direction [62]. The therapeutic potential of other OVs, such as Onyx-015 and MV-NIS, needs further investigation.

The implementation of rigorous patient evaluation, monitoring and management protocols is essential when employing OVs for immunotherapy [120]. There is a lack of research concerning the biosafety of immunotherapy, combinatorial strategies and rare subtypes of HNSCC [120]. Although generally well tolerated, OVs therapy can still induce flu-like symptoms, injection site reactions and severe irAEs, especially liver toxicity [121]. Utilizing OVs as therapeutic vectors carries the risk of inadvertent encapsulation or integration of oncogenes [114]. High immunogenicity of OVs or pre-existing neutralizing antibodies to the same serotype from previous infections can lead to rapid clearance by the host immune system, hindering viral replication and dissemination [113,115]. Furthermore, physical barriers and heterogeneity of TME, such as a dense extracellular matrix, and suppressive conditions, like limited T cell infiltration, may impede OVs spread [115].

#### Combined with tumour vaccine

Tumour vaccines represent a therapeutic strategy aimed at eliminating tumour cells by activating or enhancing the immune response against the tumour, including the expansion of antigen-specific CD4^+^ and CD8^+^ T cells [122]. These vaccines focus on tumour antigens, also known as tumour rejection antigens, which are protein/peptide sequences that are highly expressed by tumour precursors or actual tumour cells [123]. They can be divided into four principal classes of antigens: tumour-specific antigens (TSAs), tumour-associated antigens (TAAs), cancer-testis antigens (CTAs) and viral antigens involved in carcinogenesis, which are generally released into the circulation to elicit an immune response[124].

The efficacy of tumour vaccines is complementary with that of ICIs. In advanced tumours, the immunosuppressive TME may lead to rapid exhaustion of effector T cells induced by the vaccine. Under such circumstances, vaccines alone are unlikely to result in sustained tumour regression, highlighting the need to combine tumour-specific vaccines with blockade of ICs [125]. Conversely, some patients may not respond to ICIs alone, and the incorporation of tumour vaccines may stimulate an immune response against the tumour in these patients, overcoming both primary and acquired resistance to ICIs. Thus, tumour vaccines demonstrate significant potential for synergy with ICR blockade, optimizing the expansion of tumour-specific CD8^+^ T cells and preserving their functionality.

In clinical trials of HNSCC, the combination of PD-1 monoclonal antibody nivolumab and several tumour vaccines, including TG4001, MEDI0457, has achieved an improved prognosis [126]. However, current tumour vaccines are primarily combined with PD-1/PD-L1 inhibitors, and the variety of combined ICIs is too limited. To date, no vaccine delivery method has fully succeeded in achieving an effective combination of safety, efficacy and low cost [125]. The intratumoural heterogeneity of HNSCC also makes it difficult for a single vaccine to cover all tumour cells, necessitating multivalent vaccines targeting multiple antigens. This heterogeneity increases the complexity of vaccine design, as vaccines need to target the specific tumour mutation spectrum of individual patients [124]. These are severe challenges faced by vaccine efficacy, and the integration of tumour vaccines with next-generation ICIs has the potential to cope with these issues and drive significant advancements in the efficacy of tumour immunotherapy.

In the combined application of ICIs and emerging therapeutic methods, we see new hope for three treatment strategies in future clinical applications. Nanomaterials show great potential in improving drug delivery efficiency and enhancing drug penetration in the TME, OVs are a highly selective biological therapy for killing tumour cells, and tumour vaccines actively enhance the body’s immunity by activating or enhancing the body’s immune response to tumours. Notwithstanding, research in this area is currently limited, and more studies are needed to assess their safety and efficacy. Besides, whether the combination of ICIs with novel therapeutic strategies in HNSCC can assist tumour immunotherapy still requires more evidence for evaluation. In summary, the combination of nanomaterials, OVs and tumour vaccines together with ICIs has provided multi-dimensional, complementary treatment methods for tumour treatment. Future research should focus more on optimizing the combined application of these treatment methods to achieve more precise and effective tumour treatment. At the same time, a deeper understanding of the interaction mechanisms of these treatment methods will help develop more personalized and efficient treatment plans with less adverse effects, ultimately improving patient clinical outcomes.

## Conclusions and perspectives

In conclusion, the domain of immunotherapy for HNSCC is witnessing remarkable advancements, presenting a plethora of many promising opportunities to improve patient outcomes through innovative and diverse therapeutic mechanisms. Although ICIs such as PD-1/PD-L1 and CTLA-4 antibodies demonstrate significant potential, their efficacy in enhancing survival rates and diminishing morbidity remain suboptimal, as clearly demonstrated in Figure 1. The burgeoning interest in novel ICs, epitomized by the eight ICs discussed in this review, underscores the expanding horizons of this field and suggests myriad applications in HNSCC, even across a broader spectrum of malignancies.

Nonetheless, the successful translation of these emerging targets into clinical practice hinges upon a more profound understanding of their underlying mechanisms, particularly with respect to BTLA, and GITR (Table 2), as well as the identification of optimal combinatorial strategies with other ICs. On one hand, it is imperative to accelerate further investigations into the complex interactions among all constituents of the TME. This endeavour includes the examination of how ICs operate within the highly suppressive TME, which may assist to devise novel interventions that can surmount the suppressive effects of the TME, ultimately yield a thorough depiction of the comprehensive mechanisms of action of these eight ICs. On the other hand, a more nuanced exploration of novel IC molecules is of paramount importance. A series of ICIs that have yet to enter clinical research, such as GPR132, Siglec-15, ADAR1 and CD3L1 inhibitors, may exhibit more advantageous properties compared to those presently in clinical trials, which could pave the way for revolutionary therapeutic strategies capable of overcoming the limitations inherent in current ICI therapies.

**Table 2.** Clinical trials of the nine ICs around the world.

Target	NCT Number	Study status	Phases	Enrolment
PD-L2	NCT02252042	COMPLETED	PHASE3	495
	NCT02358031	COMPLETED	PHASE3	882
	NCT06081673	RECRUITING	PHASE2	72
	NCT03040999	COMPLETED	PHASE3	804
	NCT05814666	RECRUITING	PHASE2	81
	NCT03575598	COMPLETED	EARLY_PHASE1	10
	NCT06546553	RECRUITING	PHASE1	190
	NCT06285097	RECRUITING	PHASE1	140
	NCT05094804	RECRUITING	PHASE1|PHASE2	172
	NCT04634825	TERMINATED	PHASE2	62
	NCT02628535	TERMINATED	PHASE1	67
	NCT03729596	TERMINATED	PHASE1|PHASE2	143
VISTA	NCT04475523	COMPLETED	PHASE1	26
	NCT02812875	COMPLETED	PHASE1	71
	NCT05082610	RECRUITING	PHASE1	313
	NCT05864144	RECRUITING	PHASE1|PHASE2	169
BTLA	NCT04929080	COMPLETED	PHASE1|PHASE2	149
TIM-3	NCT05821751	RECRUITING	NA	40
	NCT06338657	RECRUITING	PHASE1	10
	NCT05783921	RECRUITING	PHASE1|PHASE2	60
	NCT03652077	COMPLETED	PHASE1	40
	NCT05144698	RECRUITING	PHASE1|PHASE2	22
	NCT03058289	COMPLETED	PHASE1|PHASE2	111
LAG-3	NCT04080804	RECRUITING	PHASE2	80
	NCT05821751	RECRUITING	NA	40
	NCT06338657	RECRUITING	PHASE1	10
	NCT03916627	RECRUITING	PHASE2	73
	NCT03538028	COMPLETED	PHASE1	22
	NCT03219268	COMPLETED	PHASE1	277
	NCT05144698	RECRUITING	PHASE1|PHASE2	22
	NCT03849469	COMPLETED	PHASE1	78
	NCT03058289	COMPLETED	PHASE1|PHASE2	111
TIGIT	NCT03708224	RECRUITING	PHASE2	55
	NCT05414032	RECRUITING	PHASE2	200
	NCT06338657	RECRUITING	PHASE1	10
GITR	NCT04470024	RECRUITING	PHASE1	56
	NCT04465487	RECRUITING	PHASE1	85
	NCT03126110	COMPLETED	PHASE1|PHASE2	145

*Source*: www.clinicaltrials.gov.

Furthermore, the exploration of innovative biological and chemical approaches, such as OVs, nanomaterials and tumour vaccines, presents promising avenues that could significantly augment the efficacy of the emerging ICIs. As illustrated in Figure 3, OVs arise from genetically engineered viral backbones, cancer vaccines are derived from tumour cells or their modified metabolites, and nanomedicines stem from advances in materials science. When these three emerging modalities are combined with immune-checkpoint inhibitors, a synergistic interplay may emerge in which each component compensates for the limitations of the others. We further envisage scenarios in which two or all three approaches are deployed concomitantly, anticipating that their complementary strengths will be amplified while their respective weaknesses are mitigated. The interplay between the mechanisms of these technologies may exists synergistic effects, rendering further exploration of their combined potential exceedingly promising. Building on the mechanistic landscape we have mapped, we propose four actionable pillars to accelerate next-generation ICI development. First, we have annotated the transcriptional, post-transcriptional and post-translational regulators that tune each emerging checkpoint creating a menu of druggable nodes for future trials that pair ICIs with small-molecule or RNA-based modulators. Second, by curating recent phase I–III and real-world data we reveal a clear trend towards neoadjuvant and adjuvant ICI use; we therefore champion peri-operative immunotherapy as a high-yield setting for upcoming studies, particularly in patients with intermediate-risk genetic signatures. Third, we advocate pre-treatment whole-exome or targeted sequencing to stratify TP53-null, CDKN2A-deleted, PIK3CA-mutant or 11q13-amplified tumours into basket or umbrella protocols that enable precision dosing and rational combinations. Finally, we outline synergistic platforms – therapeutic HPV vaccines for HPV^+^ lesions, OVs for inflamed TP53-mutant tumours and NP co-delivery systems for ΔNp63-high or IAP-amplified cases – that are ready for first-in-human testing. Collectively, these integrative steps should translate our bench insights into measurable clinical benefit for HNSCC patients worldwide.

However, the main limitation of this article lies in the insufficient provision of clinical data. This article is a narrative, non-systematic review of the recent literature on novel ICs. Therefore, we did not perform a systematic search or apply formal inclusion/exclusion criteria. Furthermore, given the pre-clinical emphasis of this review, patient-level clinical end-points such as progression-free survival, OS or treatment-related adverse events are not systematically synthesized; the reader is referred to recent clinical reviews for these data.

To encapsulate, the in-depth exploration of these ICI-related immunotherapeutic technologies, when integrated with specific clinical contexts, will significantly propel the advancement of personalized treatment and precision medicine, equipping us with increasingly effective tools in the relentless battle against a myriad of complex diseases. Therefore, it is essential to intensify research efforts directed towards clinical trial outcomes, elucidation of mechanisms of action, and integrating emerging ICIs, alongside the development of novel technologies and personalized treatment strategies, to optimize immunotherapy for patients afflicted with HNSCC.

## Data Availability

Data sharing is not applicable to this article, as no new data were created or analyzed in this study.
